# Splenectomy versus Partial Splenic Embolization for Massive Splenomegaly Secondary to Hepatitis B-Related Liver Cirrhosis: A Case-Control Study

**DOI:** 10.1155/2016/3471626

**Published:** 2016-06-22

**Authors:** Shoufei Jiao, Hongxing Chen, Youlong Wang, Jiye Zhu, Jingwang Tan, Jie Gao

**Affiliations:** ^1^Department of Hepatobiliary Surgery, Peking University People's Hospital, Beijing 100044, China; ^2^Chinese PLA Medical School and Chinese PLA General Hospital, Beijing 100853, China

## Abstract

*Background.* Both splenectomy (SP) and partial splenic embolization (PSE) are used to treat massive splenomegaly (MSM) secondary to hepatitis B-related liver cirrhosis (HB-LC). This retrospective case-control study was conducted to compare the effects of SP and PSE on these patients.* Methods.* From July 2004 to January 2012, patients with MSM secondary to HB-LC who underwent SP or PSE were 1 : 1 : 1 matched with similar nonsurgery patients, respectively. Intraoperative situation, hematological indices, liver function, HBV DNA level, HBeAg seroconversion rate, morbidity, and mortality at 6 months postoperatively were compared.* Results.* Operative time, estimated blood loss, blood transfusion rate, severe pain, postoperative stay, and portal vein thrombosis (PVT) rate in the PSE group were significantly superior to the SP group, although SP and PSE were similar in liver function improvement, HBV suppression, morbidity, and mortality at 6 months postoperatively, and SP even improved WBC and PLT counts higher than PSE.* Conclusion.* Both SP and PSE are effective in improving liver function, increasing WBC and PLT counts, and suppressing replication of HBV for MSM secondary to HB-LC. Although postoperative improvement in WBC and PLT counts by SP can be higher than PSE, PSE is simple and minimally invasive and has a lower incidence of PVT.

## 1. Introduction

Hepatitis B is highly prevalent in China and frequently associated with liver cirrhosis and portal hypertension (PH) that often cause splenomegaly [1, 2]. SP is the most common surgical treatment for MSM secondary to HB-LC [3]. It has been generally agreed that SP is performed to control esophageal varices bleeding or as a modality in order to reverse severe thrombocytopenia and leukopenia. However, there are some risks associated with SP [4], such as hemorrhage, pulmonary atelectasis, pneumonia, pleural effusion, subphrenic abscess, gastric ileus, venous thrombosis, overwhelming postoperative infection (OPSI), and atherosclerosis. Alternatively, PSE could be an option that may have some advantages over SP in some instances [5]. Little is known about the comparison of SP and PSE on MSM secondary to HB-LC. This retrospective case-control study aimed to characterize the effects of PSE, in comparison with SP, on hematological indices, liver function, anti-hepatitis B virus, and PVT incidence in patients with MSM secondary to HB-LC.

## 2. Methods

### 2.1. Study Design

**Figure 1:**
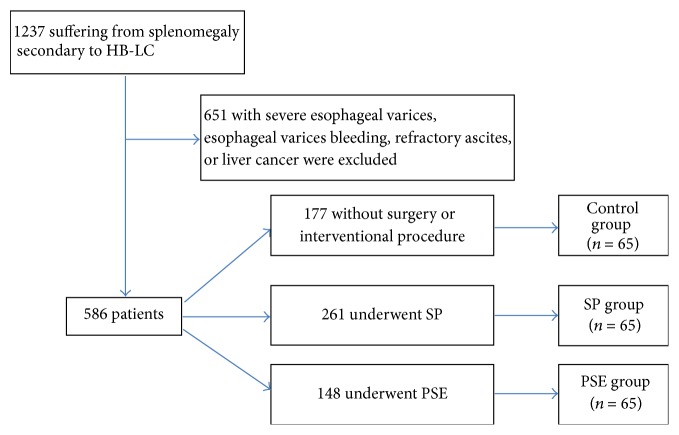
Flow diagram outlining the study design.

From July 2004 to January 2012, there were 1237 patients suffering from splenomegaly secondary to HB-LC who have been treated in our two institutes. Within these patients, 651 patients with severe esophageal varices, esophageal varices bleeding, refractory ascites, or liver cancer were initially excluded. Among the remaining 586 patients, 177 patients with mild hypersplenism (PLT > 6.0 × 109/L) were treated without surgery or interventional procedure, 148 patients underwent PSE, and 261 patients underwent SP. Within those without surgery or interventional procedure, 65 patients with enlarged spleen (20–27 cm in craniocaudal length and 1000 g–2500 g in weight) were assigned as the control group. Using a 1 : 1 : 1 case-control ratio, these 65 patients were randomly matched to patients who underwent PSE (PSE group) or SP (SP group) (Figure 1). The matching criteria included Child-Pugh grade, gender, age, serum HBV DNA level, antiviral therapy, spleen weight, esophageal varices degree, indocyanine green 15 min retention rate (ICG R15), comorbidities rate, and ASA grade.

All patients were subjected to detailed history, thorough physical examination, laboratory investigations (including bone marrow aspiration), abdominal ultrasonography, color-coded duplex scanning of the portal circulation, upper gastrointestinal endoscopy, and abdominal computed tomography (CT) scans with oral and intravenous contrast and diagnosed by liver biopsy. All patients were provided with antiviral therapy if serum HBV DNA tests were positive. Liver-protective drugs were administered to patients with hepatic insufficiency. For splenomegaly, there were three treatment modalities: SP, PSE, or conservative treatment depending on PLT count and Child-Pugh grade with patients' consent. The spleen size was accurately measured before and after procedure by abdominal CT or ultrasound according to the recognized definition [6].

All patients gave informed consent and the study was authorized by the Hospital's Ethics Committee. Patient confidentiality was preserved according to the guidelines for studies of human subjects.

### 2.2. PSE Procedure

Under strict aseptic condition, PSE was performed according to a standard approach [5, 7]. Briefly, the femoral artery was punctured by a 5.0 French catheter (Cook, Bloomington, USA; Terumo, Tokyo, Japan) via the Seldinger approach. Preliminary splenic arterial angiography was obtained to determine the configuration of splenic artery and the location of pancreatic branches. The tip of the catheter was placed as distal as possible at the hilus of the spleen in order to avoid ectopic embolization, and embolization was performed using embolic agent suspended in an antibiotic solution (gentamicin sulphate 16 mg) and contrast medium. The splenic infarction ratio was set at 50–70% [8]. During embolization, small amounts of contrast material were periodically injected through the catheter to monitor the flow distribution in the spleen. Immediately after each particle injection, postembolization angiography was performed and the infarction rate was calculated. Once a 50–70% ablation of splenic parenchyma was achieved, catheter was irrigated with saline and removed. The site of puncture was compressed for about 15 minutes.

Post-PSE supportive care included appropriate hydroelectrolytic infusion, systemic prophylaxis with intravenous antibiotics, cefoperazone (1 g/12 h) for 5 days, and adapted analgesic treatment with nonsteroidal anti-inflammatory drugs or morphine. All patients remained in hospital until postembolization syndrome or any other significant complications disappeared. The precise infarction rate was calculated on CT examinations 2 weeks after PSE. Routine abdominal ultrasonography was performed at 30 day after PSE to exclude PVT.

The embolic agent used in this study was polyvinyl alcohol (PVA) in contour particles (Boston Scientific, Natick, MA, USA). Particle sizes are in the range of 355–500 *μ*m exclusively. Particles smaller than this range are not recommended considering the possibility of a more intense necrosis. For most of our patients, one vial of PVA was sufficient to embolize at least 50% of the spleen parenchyma.

### 2.3. SP Procedure

The operation was performed under general anesthesia with open or laparoscopy approach according to standard procedures [3, 9]. All the operations were performed by the same experienced surgeon. SP was carried out immediately after opening and exploring the abdomen. In laparoscopic SP, the splenic artery was divided and ligated firstly in order to avoid massive hemorrhage. Subsequently, the splenic hilum was transected. Lateral position facilitates the dissection of perisplenic ligaments and hilus lienis structures due to gravity. Additionally, the operating table can be tilted to obtain appropriate patient's position at any time during surgery. The most common cause of convert from laparoscopic to open SP is unmanageable bleeding. Patients received pharmacologic or mechanical thromboprophylaxis after SP or PSE according to the risk for VTE and bleeding complications [10].

### 2.4. Antiviral Therapy and Follow-Up

Three groups were provided with standard antiviral agents, including lamivudine, entecavir, or adefovir. All patients were followed up at 1 m, 3 m, and 6 m postoperatively. At each visit, a detailed history, physical examination, blood routine examination, liver function, serum HBV DNA level, HBeAg, ultrasound examination for PVT, and ICG R15 were obtained. If PVT was suspected in patients according to the routine ultrasound, clinical signs, or symptoms (such as abdominal distension, diarrhea, fever, abdominal discomfort, leukocytosis, and nausea), abdominal CT scans were subsequently performed to confirm this.

### 2.5. Statistical Analysis

Continuous variables were expressed as mean ± standard deviation (SD) and analyzed using *t*-test. Qualitative variables were presented as number and percent. The chi-squared or Fischer's exact test was used for comparison between groups as appropriate. Statistical analyses were performed using SPSS software (version 13.0; SPSS Inc., Chicago, IL). A value of *P* < 0.05 was considered statistically significant.

## 3. Results

### 3.1. Preoperative Characteristics and Operative Outcomes

Except for WBC and PLT counts, there were no significant differences in Child-Pugh grade, gender, age, serum HBV DNA level, antiviral therapy, spleen weight, esophageal varices degree, ICG R15, comorbidity rate, and ASA grade between these three groups (Table 1, *P* > 0.05). Notably, operative time, estimated blood loss, blood transfusion rate, severe pain, postoperative stay, and PVT rate in the PSE group were significantly lower than the SP group (*P* < 0.05). Only one patient died of esophageal varices bleeding secondary to PVT in the SP group, and the total complication rate is similar between the SP and PSE groups (Table 2).

### 3.2. Hematological Indices

**Figure 2:**
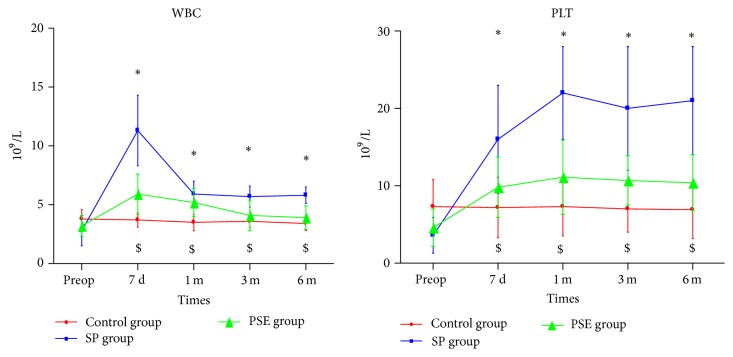
The preoperative and serial postoperative measurements of WBC and PLT counts. (*∗* indicates significant difference compared with the baseline in the PSE group, *P* < 0.05; $ indicates significant difference compared with the baseline in the SP group, *P* < 0.05.)

The preoperative and serial postoperative measurements of WBC and PLT counts are shown in Figure 2. Postoperative WBC and PLT counts rose higher in the SP and PSE groups compared with their preoperative results (*P* < 0.05), whereas there was no significant difference in the control group who have not received any drugs for improving WBC and PLT counts (*P* > 0.05). The postoperative increases of WBC and PLT counts in the SP group were higher than those in the PSE group (*P* < 0.05).

### 3.3. Liver Function

**Figure 3:**
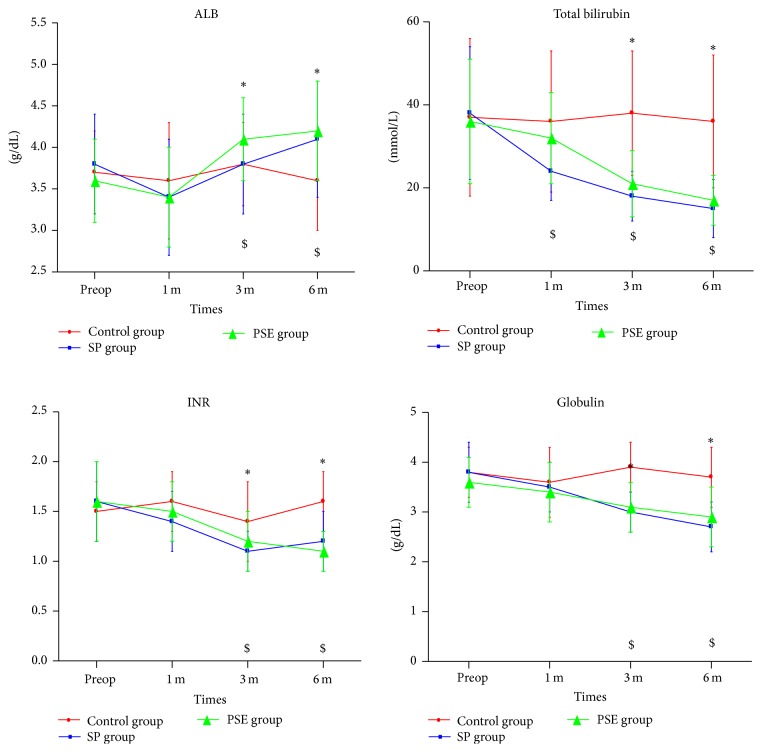
Serum albumin (ALB), total bilirubin, international normalized ratio (INR), and globulin before and after the procedure. (*∗* indicates significant difference compared with the baseline in the PSE group, *P* < 0.05; $ indicates significant difference compared with the baseline in the SP group, *P* < 0.05.)

The levels of serum albumin, total bilirubin, international normalized ratio (INR), globulin, and ICG R15 before and after the procedures are summarized in Table 3 and Figure 3. At 3 m and 6 m postoperatively, patients in both SP and PSE groups had significantly increased albumin (*P* < 0.05) and sharply decreased TB, INR, and globulin (*P* < 0.05). In contrast, these values in control patients remained unchanged comparing to preoperative levels (*P* > 0.05). Child-Pugh grade and ICG R15 at 6 m postoperatively were also improved significantly in the SP and PSE groups when compared with their preoperative state, as well as the values of the control group (*P* < 0.05). Overall, liver function improvement was indistinguishable between the SP and PSE groups (*P* > 0.05).

### 3.4. HBV DNA Level and HBeAg Seroconversion Rate

HBV DNA levels and HBeAg seroconversion rates before and after the procedures are shown in Table 3. Postoperative HBV DNA levels decreased, and HBeAg seroconversion rates increased significantly in all patients (*P* < 0.05). Specifically, comparing to controls, HBV DNA levels have declined more in patients receiving SP or PSE (*P* < 0.05).

### 3.5. PVT

The incidences of PVT among the three groups are displayed in Table 3. The incidence of PVT in the SP group (29.2%) was significantly higher than that in both control (0%) and PSE groups (3.0%) (*P* < 0.05).

## 4. Discussion

### 4.1. Hematological Indices

Splenomegaly is a frequent finding in patients with HB-LC [1, 2, 11]. However, splenomegaly, even MSM secondary to HB-LC, is not considered as a surgical indication unless accompanied with severe hypersplenism or esophageal varices bleeding.

Hypersplenism may cause decreased blood cell counts mainly through five mechanisms [12]: (1) proportional decrease in peripheral blood cells due to the abnormal expansion of cell storage in the enlarged spleen; (2) enhanced phagocytosis of blood cells by monocyte and macrophage during hypersplenism; (3) myelosuppression induced by hepatitis B virus and hypersplenism; (4) autoantibody to blood cells produced by spleen; (5) esophageal varices bleeding that is often accompanied by hypersplenism. Accordingly, it has been known that SP and PSE, both of which can lead to regression of splenomegaly, could raise WBC and PLT counts. Consistently in our study, postoperative WBC and PLT counts rose significantly in patients who underwent SP or PSE (*P* < 0.05). Interestingly, the increase is more obvious in SP-treated patients, which may be because of residual spleen parenchyma in PSE.

### 4.2. Liver Function

It has been well established that SP and PSE could alleviate portal vein pressure and improve liver function [13]. However, few studies have examined whether or not these effects may persist for more than six months. Ushitora et al. [14] have reported that SP and PSE could have a long-term effect on liver function, in which HB-LC patients were not evaluated. The assessments performed in our study suggest that SP and PSE have a lasting improvement in liver function also on HB-LC for at least six months.

The mechanism by which SP and PSE could improve liver function has not yet been elucidated. Decreasing portal vein pressure may be beneficial to prevent pathological bacterial translocation to the liver. In addition, the decrease of splenic vein flow leads to a compensatory increase of hepatic artery and mesenteric vein flow, resulting in more nutrient-rich blood to the liver [15]. Moreover, an immunologic mechanism has been suggested by some studies [14, 16, 17]. For instance, it is possible that increased WBC could inhibit the replication of HBV and hence protect liver function, and antiviral therapy might indirectly contribute to the improvement of liver function as well.

### 4.3. HBV DNA Level and HBeAg Seroconversion Rate

Up to now, there are few studies concerning the impact of SP or PSE on HBV DNA level. We found that SP and PSE could reduce HBV DNA level, which may facilitate long-term improvement of liver function and possible prevention of disease progression. The nature and underlying mechanism of this intriguing finding is still unclear at the moment and certainly deserves further study in the future.

### 4.4. PVT

Previous studies have reported that the incidence of PVT after SP was between 8% and 50% [18]. On top of that, a large review of 37012 autopsies has shown that the odds of fatal pulmonary embolism in persons with SP were 5-fold higher than matched controls who had not undergone SP [19]. Thomsen et al. [20] examined SP patients up to a year after surgery and identified that the adjusted relative risk of any venous thromboembolism in these patients is 3.4- or 3.2-fold as the general population and appendectomized patients, respectively. The incidences of PVT in our study were 29.2% (19/65) after SP and 3.0% (2/65) after PSE. It is possible that PVT after SP is associated with the increased amount and enhanced aggregation competence of platelet after SP. In addition, a higher plasma viscosity after SP can be attributed to increased blood cells and greater rigidity of erythrocytes [21]. The size of spleen before SP is likely to be associated with the incidence of PVT. Still blood in the stump of portal vein after SP also predisposes to PVT, which may explain the less PVT in PSE than SP [22].

In our study, there was one occurrence of PVT in a SP patient who died of gastrointestinal bleeding. Although most PVT remain asymptomatic and could recover well, this complication could be fatal if not timely treated, especially in the early stage. PVT after SP should be suspected whenever patients have abdominal pain, ileus, or fever. Color Doppler ultrasonography has a sensitivity of 93% and a specificity of 99% for diagnosing PVT, and routine Doppler ultrasonography after SP might be beneficial for early diagnosis. If diagnosed, prompt anticoagulation therapy can help to restore normal flow of portal vein [22]. Kawanaka et al. [23] have described the risk stratification of PVT after SP and reported that the administration of antithrombin III and danaparoid sodium could reduce the incidence of PVT.

### 4.5. OPSI

Besides PVT, OPSI is another complication worth noting associated with SP. The mortality rate of OPSI is 38–70% despite adequate treatment, and most deaths occur within 24 h–48 h [24]. Thus, the Society for Surgery of the Alimentary Tract recommended that SP can only be performed after trauma when blood loss volume was more than 1000 mL, transfusion volume was more than 2 units, hemodynamics was unstable, or progressive hemorrhage was hinted. Otherwise, hemostatic, ligation and partial angioembolization of the splenic artery, simple splenorrhaphy, or partial SP was preferable [25]. When SP is compelled under certain circumstances, autotransplantation of splenic tissue in omental pockets might reserve sufficient splenic function [26]. It is estimated that at least 30 mL of splenic tissue is needed for return of splenic function after SP [27].

Compared with SP, several notable merits in PSE methods were documented in the present study: (1) PSE is minimally invasive, thus reducing operative time, blood loss, severe pain, and postoperative stay; (2) PSE has low incidence of PVT; (3) PSE preserves moderate splenic parenchyma to avoid OPSI; (4) PSE can also reverse hypersplenism, improve liver function, and even reduce HBV DNA level.

To our surprise, patients in the SP group experienced more severe pain than the PSE patients, which is different from the published data [5, 28]. The distinct choices of drug regimen may lead to such unexpected results of pain perception in patients, since all PSE patients used nonsteroidal anti-inflammatory drugs or morphine for analgesia immediately after PSE, while only a few SP patients volunteered to choose postoperative autocontrolled analgesic pump. As a result, only 9 of 65 PSE patients felt severe pain, and 25 of 65 PS patients felt severe pain.

Finally, the postoperative side-effects of PSE may include (1) postembolization syndrome, such as fever, nausea/vomiting, and spleen area pain; (2) ectopic embolization; (3) left pleural effusion; (4) splenic abscess; and (5) recurrence of MSM. Child-Pugh class C and splenic infarct volume >540 mL are independent risk factors for complications after PSE. A partitioned and repeated PSE might therefore be a safer option in Child-Pugh class C patients to avoid the complications [29]. Although most complications after PSE can be alleviated by conservative treatment, percutaneous puncture and drainage of the abscess or SP is the final solution to splenic abscess. For patients with recurrent MSM, repeated PSE or SP is required.

## 5. Conclusion

In a summary, our study demonstrated that both SP and PSE could improve liver function, increase WBC and PLT counts, and suppress replication of HBV in patients with MSM secondary to HB-LC. These data indicate possible prevention of disease progression by both procedures. In comparison with SP that can raise more WBC and PLT counts, PSE is simple and minimally invasive and has a low incidence of PVT. PSE can be an effective option for patients who are not surgical candidates and for whom SP is contraindicated. Although our patient cohort was in a small number and mainly restricted to Child-Pugh class A, the findings suggest important differences between SP and PSE for MSM secondary to HB-LC. With longer follow-up and the additional measurement of portal vein pressure, a prospective randomized control trial based on our study would be potentially important to further identify the difference between SP and PSE with respect to liver function, replication of HBV, and long-term prognosis.

## Figures and Tables

**Table 1:** Background characteristics of enrolled patients.

Parameters	Control group (*n* = 65)	SP group (*n* = 65)	PSE group (*n* = 65)	*P* value
Child-Pugh grade (A : B : C)	54 : 11 : 0	54 : 11 : 0	54 : 11 : 0	1.0000
Gender (M : F)	43 : 22	45 : 20	44 : 21	0.9321
Age (y)	45.3 ± 7.1	46.1 ± 8.7	46.4 ± 9.4	0.7771
WBC count (/mm^3^)	3.75 ± 0.88	2.81 ± 1.02	3.29 ± 0.95	<0.001
PLT count (10^3^/mm^3^)	7.3 ± 2.5	3.6 ± 2.7	4.5 ± 2.4	<0.001
HBV DNA level (log_10_)	4.27 ± 0.91	4.16 ± 0.85	4.34 ± 0.88	0.5025
Antiviral protocol				0.9806
Lamivudine	29	28	28	
Adefovir plus lamivudine	6	5	5	
Entecavir plus lamivudine	2	2	1	
Spleen weight (g)	1376 ± 241	1440 ± 263	1416 ± 219	0.3146
Esophageal varices degree (mild : no)	21 : 44	24 : 41	26 : 39	0.6565
ICG R15 (%)	19.2 ± 8.4	18.4 ± 9.1	20.3 ± 10.7	0.5167
Comorbidities	11	10	8	0.7531
Diabetes	3	3	2	
Hypertension	5	4	3	
COPD	1	0	2	
Others	2	3	1	
ASA grade (I : II : III)	41 : 16 : 8	44 : 17 : 4	38 : 19 : 8	0.6793

ICG R15, indocyanine green 15 min retention rate.

**Table 2:** Intraoperative and postoperative results.

Parameters	SP group (*n* = 65)	PSE group (*n* = 65)	*P* value
Operative time (min)	107 ± 12	21.33 ± 4.16	<0.05
Estimated blood loss (mL)	610 ± 136	0.5	<0.001
Rate of blood transfusion [*n* (%)]	7 (10.7%)	0	<0.05
Severe pain [*n* (%)]	25 (38.5%)	9 (13.8%)	<0.05
Complications [total (%)]	17 (26.2%)	15 (23.1%)	0.6839
Intra-abdominal abscess	1	0	
Ascites	10	6	
Internal bleeding	1	0	
Left pleural effusion	0	8	
Atelectasis	4	1	
Pneumonia	1	0	
Postoperative stay (d)	10.6 ± 2.3	9.5 ± 2.1	<0.01
PVT	19 (29.2%)	2 (3.0%)	<0.001
Mortality [*n* (%)]	1 (1.5%)	0	1.0000

**Table 3:** Short-term outcomes (6 m) among the three groups.

Group	Time	HBV DNA level (log_10_)	HBeAg positive [*n* (%)]	ICG R15 (%)	Child (A : B : C)	PVT [*n* (%)]
Control group (*n* = 65)	Baseline	4.27 ± 0.91	37/37 (100%)	19.2 ± 8.4	54 : 11 : 0	0
	After 6 m	1.96 ± 0.83^*∗*^	29/37 (78.4%)^*∗*^	18.8 ± 8.9	55 : 10 : 0	0

SP group (*n* = 65)	Baseline	4.16 ± 0.85	35/35 (100%)	18.4 ± 9.1	54 : 11 : 0	0
	Postop (6 m)	1.14 ± 1.05^*∗*#^	24/35 (68.6%)^*∗*^	14.8 ± 8.5^*∗*#^	62 : 3 : 0^*∗*#^	19 (29.2%)^*∗*#^

PSE group (*n* = 65)	Baseline	4.34 ± 0.88	34/34 (100%)	20.3 ± 10.7	54 : 11 : 0	0
	Postop (6 m)	0.97 ± 1.02^*∗*#^	21/34 (61.7%)^*∗*^	16.4 ± 9.3^*∗*#^	63 : 2 : 0^*∗*#^	2 (3.0%)^&^

^*∗*^ *P* < 0.05 is considered statistically significant compared with the baseline, ^#^ *P* < 0.05 is considered statistically significant compared with the control group, and ^&^ *P* < 0.05 is considered statistically significant compared with the SP group.
